# Analysis of the Efficacy and Safety of Endoscopic Retrograde Cholangiopancreatography in Children With Symptomatic Pancreas Divisum

**DOI:** 10.3389/fped.2021.761331

**Published:** 2021-11-02

**Authors:** Guixian Pan, Kaihua Yang, Biao Gong, Zhaohui Deng

**Affiliations:** ^1^Department of Gastroenterology, Shanghai Children's Medical Center, Shanghai Jiao Tong University School of Medicine, Shanghai, China; ^2^Department of Gastroenterology, Shanghai Shuguang Hospital, Shanghai University of Traditional Chinese Medicine, Shanghai, China

**Keywords:** endoscopic retrograde cholangiopancreatography, pancreas divisum, post-ERCP pancreatitis, efficacy and safety, children

## Abstract

**Background:** Endoscopic retrograde cholangiopancreatography (ERCP) has been increasingly performed in children with symptomatic pancreas divisum (PD).

**Aim:** To investigate the safety and efficacy of ERCP in the treatment of children with symptomatic PD.

**Methods:** We performed a retrospective analysis on children with PD who were treated with ERCP at Shanghai Children's Medical Center between June 2015 and May 2020. Pertinent patient, clinical and procedural data were collected to assess the therapeutic effects and identify the risk factors for post-ERCP pancreatitis (PEP).

**Results:** Overall, 114 ERCPs were performed in 46 children with PD. With a median follow-up of 28.5 months (12–71 months), 40 (87.0%) children achieved clinical remission, the median number of acute pancreatitis episodes decreased from four times per year pre-operatively to once per year post-operatively (*P* < 0.001), and the nutritional score improved post-operatively (*P* = 0.004). The incidence of PEP was 7.9%, and female sex, stone extraction, and gene mutations were identified as possible risk factors for PEP on univariate analysis. However, there was no statistical significance on multivariate analysis (*P* > 0.05).

**Conclusion:** Therapeutic ERCP is an effective and safe intervention for children with symptomatic PD.

## Introduction

Pancreatic divisum (PD) is the most common congenital variant of the pancreatic duct during development. It results from the failure of ventral and dorsal pancreatic duct fusion during embryonic development, and it is a risk factor for recurrent and chronic pancreatitis (CP). PD is classified into three types depending on its clinical presentation of acute recurrent pancreatitis (ARP), CP, and chronic abdominal pain.

Endoscopic retrograde cholangiopancreatography (ERCP) is the primary treatment for patients with symptomatic PD. Its efficacy and safety have been demonstrated in adults who have undergone operations such as sphincterotomy, papillary dilation, and pancreatic duct stenting (1). Despite the increasing use of ERCP among pediatric populations, information is lacking on ERCP for PD in children, and there are few studies on its efficacy and safety.

Further clinical studies are needed to assess whether children can benefit from ERCP, and how post-ERCP complications can affect children. In this study, we retrospectively analyzed the clinical data of 46 children with symptomatic PD treated with ERCP to assess the outcomes and complications and to analyze the risk factors for post-ERCP pancreatitis.

## Materials and Methods

### Patient Information

This study was approved by the Institutional Review Board of Shanghai Children's Medical Center. We retrospectively analyzed the sex, age, nutritional status, genetic analysis, ERCP procedural technique, post-operative complications and other clinical data of 46 children with symptomatic PD treated with ERCP in Shanghai Children's Medical Center from June 2015 to May 2020. The number of acute pancreatitis episodes and nutritional status of the subjects pre- and post-ERCP were also followed up long-term *via* telephone calls.

### Inclusion and Exclusion Criteria

The inclusion criteria were as follows: (1) age <18 years, and (2) children whose ERCP examination was consistent with the diagnosis of PD and who subsequently underwent treatment.

The exclusion criteria were as follows: (1) patients with failed intraoperative maneuvers; (2) patients who were lost to post-operation follow-up; and (3) patients with a previous history of ERCP management.

The diagnostic criteria for PD under ERCP were as follow: (1) the ventral pancreatic duct is observed like a rat's tail or not visualized on major papillary cannulation angiography; (2) the dorsal pancreatic duct, which runs through the entire pancreas, is visualized only through minor papillary cannulation and is not connected to the thin and short pancreatic duct of the major papilla; and (3) a thin traffic branch between the ventral and dorsal pancreatic duct is termed an incomplete PD (2).

### Grouping

The patients were assigned to the PEP or non-PEP groups according to whether or not pancreatitis occurred after ERCP. PD was classified into ARP or CP according to the clinical presentation. The diagnosis of CP was based on the criteria developed by the International Study Group of Pediatric Pancreatitis: In search for a cure (INSPPIRE). ARP is defined as at least two episodes of abdominal pain with 3-fold elevation of serum amylase or lipase from normal levels and without changes in CP on imaging.

### ERCP Procedures

**Figure 1:**
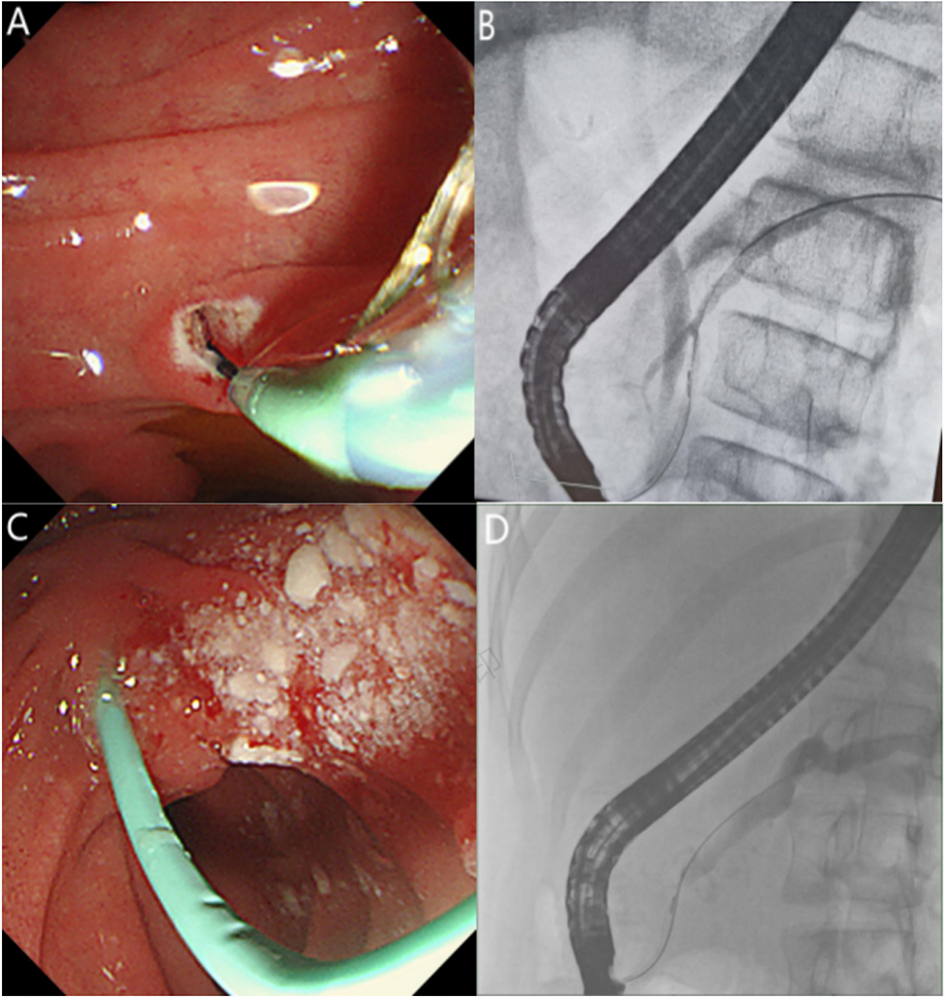
**(A)** Endoscopic view of pancreatic sphincterotomy through the minor papilla. **(B)** Fluoroscopic view of the endoscopic retrograde cholangiopancreatography showing a small ventral pancreatic duct and large dorsal pancreatic duct in incomplete pancreas divisum. **(C)** Endoscopic view showing that a pancreatic stent was placed through the minor papilla after stone extraction. **(D)** Fluoroscopic view of pancreatic duct dilation and stricture.

Prior to ERCP, each child's guardian signed a written informed consent for the procedure. There were no medications used for PEP prevention before the ERCP procedure. All procedures were performed by the same experienced endoscopist who had performed >30,000 ERCPs. The children underwent ERCP in a prone position under general anesthesia using a standard pediatric duodenoscope (JF-240, Olympus, Tokyo, Japan), while vital signs were continuously monitored. Therapeutic maneuvers were selected during the operation according to pancreatic imaging findings, including endoscopic major and/or minor papillary sphincterotomy, placement of a pancreatic duct stent or nasopancreatic duct *via* the major or minor papilla, balloon and bougie dilation, and stone extraction. The size and length of the inserted stent were determined by the degree and location of the pancreatic duct stenosis, and the pancreatic duct stent was replaced every 3–6 months or as necessary, based on the clinical symptoms of the children and the improvement of the pancreatic duct structure under ERCP. However, a pancreatic duct stent was inserted in patients with ARP for prevention then removed within 2 weeks after ERCP. Figures 1A,C shows the most common ERCP procedures of minor papillary sphincterotomy and duct stent. The fluoroscopic view of pancreatic duct is shown in Figures 1B,D. Post-ERCP complications were assessed by monitoring the serum amylase and lipase levels, pancreatic ultrasound and post-operative abdominal pain 24 h after ERCP. Procedural complications were treated using the standard medical management, and further evaluation and treatment were required for children with severe disease. The patients who met discharge criteria (i.e., tolerated oral intake, no abdominal pain, and had normal blood amylase levels) were discharged. Otherwise, they were admitted for further evaluation.

### Assessment Indicators and Follow-Up

The data of the first ERCP was regarded as the starting point of follow-up, while the end of follow-up was set on May 31, 2021. Primary observations were clinical remission rate and post-ERCP improvements. The clinical remission rate was defined as a reduction of over 50% in the number of acute pancreatitis episodes annually after follow-up relative to pre-treatment frequency. The post-ERCP improvements were as follows: (1) nutritional status: The WHO Anthro software (children's version) was used to calculate the BMI-for-age Z score (BAZ) to assess the nutritional status of the children. Comparing the BAZ before surgery with that 1 year post-primary ERCP, a BAZ below 2 standard deviations was defined as malnutrition; (2) number of acute pancreatitis episodes: the pre- and post-ERCP numbers of acute pancreatitis annually were compared.

The secondary observations were post-ERCP complications, among which PEP was the most common. PEP was defined as new or worsening abdominal pain from acute pancreatitis and at least a three-fold elevation in serum amylase levels 24 h after the procedure. The severity of PEP was classified according to Cotton's criteria (3) as follows: mild, requiring additional hospitalization for 1–3 d; moderate, requiring additional hospitalization for 4-10 d; and severe, requiring hospitalization for >10 d, as well as hemorrhagic pancreatitis and pseudocysts.

For the PEP risk factor analysis, we analyzed the effects of patient factors (e.g., sex, age, CP, and genetic mutation status) and operative factors (e.g., placement of pancreatic duct stent, stone extraction, dilation, and sphincterotomy) on post-operative pancreatitis.

### Statistical Methods

The analysis was performed using SPSS 25.0 statistical software. The quantiative data were analyzed through normality tests, with normally and non-normally distributed measures expressed as mean ± standard deviation (x ± s) and median, respectively. They were analyzed using non-parametric tests. Count data are expressed as percentages (%) and were analyzed using the chi-square test or Fisher's exact-test when the actual frequency was <5. Binary logistic regression was applied to analyze the risk factors for PEP. Statistical significance was set at *P* < 0.05.

## Results

### Basic Patient Information

Forty-seven pediatric patients with PD were included in this study. As one patient was lost to post-operative follow-up after failure of intraoperative maneuvers, the remaining 46 patients were included in the final analysis (Table 1). Forty-six pediatric patients were diagnosed with PD at the first ERCP, including 22 (47.8%) females and 24 (52.2%) males. The median age of patients at PD onset was 9 years old. There were 27 cases of incomplete PD and 19 cases of complete PD in our study. All subjects had varying degrees of abdominal pain, and 19 patients had accompanying nausea and vomiting during the course of the disease. Thirteen (39.13%) and 33 (60.87%) patients had ARP and CP, respectively. Genetic testing was performed in 26 patients, which showed 15 cases of gene mutations. Two types of mutations, PRSS1 and SPINK1, were detected in four cases (15.4%) and 11 cases (42.3%), respectively. All patients had no history of smoking, drinking, or hypertriglyceridemia.

**Table 1:** Baseline characteristics of 46 pediatric patients with pancreas divisum.

	**No**.	**%**
**Diagnosis**		
ARP	13	28.3
CP	33	71.7
**Sex**		
Male	24	52.2
Female	22	47.8
**Genetic mutations**		
SPINK1	11	23.9
PRSS1	4	8.7
Negative	11	23.9
Untested	20	43.5
Hypertriglyceridemia, smoking, drinking	0	0.0
Median age at initial ERCP (range), yr	9 (1–14)	–
Complete PD	19	41.3
Incomplete PD	27	58.7

*ARP, acute recurrent pancreatitis; CP, chronic pancreatitis; PRSS1, cationic trypsinogen; SPINK1, serine protease inhibitor Kazal-type 1; PD, pancreas divisum*.

### ERCP Procedures

A total of 114 ERCP treatments were performed in 46 patients (Table 2). Sphincterotomy was performed in 40 cases, including 20 minor papillotomies, 11 major papillotomies, and 9 concomitant minor and major papillotomies. Stone extraction was performed in 68 cases, major papilla stents and minor papilla stents were placed in 22 (19.3%) and 73 (64%) cases, respectively. Pancreatic duct stricture dilation was performed by balloon manipulation and bougie manipulation in 19 (16.7%) and 25 (21.9%) cases, respectively. Operation of the major papillary was mainly performed in patients with incomplete PD (Figure 1B). In the case of incomplete PD, sphincterotomy and pancreatic duct stent placement *via* the major papilla was performed in 20 and 22 cases, respectively.

**Table 2:** Therapeutic details of 114 procedures in 46 pediatric patients with pancreas divisum.

	**Procedures**	**%**
**Sphincterotomy**		
Major papillotomy	11	9.6
Minor papillotomy	20	17.5
Both	9	7.9
**Dorsal duct stent placement**		
Major papilla	22	19.3
Minor papilla	73	64.0
**Dilation**		
Balloon dilation	19	16.7
Bougie dilation	25	21.9
Stone extraction	68	59.6

*This table is designed on the number of procedures*.

### Post-ERCP Complications and Risk Factors Analysis

PEP was identified in nine (7.9%) of 114 ERCPs performed, of which one was mild and eight were moderate in severity (Table 3). They were discharged after improvement with routine acid and enzyme suppression therapy. The incidence of PEP was 7.9%, and in the univariate analysis for the occurrence of PEP, female sex (*P* = 0.001), stone extraction (*P* = 0.039), and genetic mutation (*P* = 0.007) were found to be risk factors for PEP (Table 4). However, they were not statistically significant (*P* > 0.05) on multivariate analysis (Table 5).

**Table 3:** Prevalence and severity of PEP.

	**No**.	**(%)**
**Severity of PEP**		
**PEP**		
Mild	1	0.9
Moderate	8	7.0
Severe	0	0.0
Total	9	7.9

*PEP, post-endoscopic retrograde cholangiopancreatography pancreatitis*.

**Table 4:** Long-term outcomes of 46 pediatric patients.

	**AP**		***P***	**BAZ**		***P***	**Remission**
	**Pre-ERCP**	**Post-ERCP**		**Pre-ERCP**	**Post-ERCP**		
All	4 (1~>10)	1 (1~3)	0.000	−0.42 ± 1.15	0.04 ± 1.16	0.004	40 (87.0%)
CP	4 (1~>10)	1 (1~3)	0.000	−0.57 ± 1.24	0.04 ± 1.24	0.003	29 (87.9%)

*AP, the number of episodes of acute pancreatitis per year; BAZ, BMI for age z score*.

*ERCP, Endoscopic retrograde cholangiopancreatography; CP, chronic pancrestitis*.

**Table 5:** Univariate analysis of factors associated with PEP development.

	**PEP(%)**	**No PEP(%)**	***P***
Patients (*n* = 114)	9 (7.9)	105 (92.1)	
Female(%)	0 (0.0)	58 (55.2)	0.001^*^
Age (Y)			0.268
<3	0 (0.0)	1 (1.0)	
3–6	0 (0.0)	21 (20.0)	
>6	9 (100)	83 (79)	
Stent placement	9 (100)	86 (81.9)	0.162
Stone extraction	8 (88.9)	56 (53.3)	0.039^*^
Dilation	4 (44.4)	40 (38.1)	0.707
Sphincterotomy	7 (77.8)	88 (83.8)	1.000
Chronic pancreatitis	8 (88.9)	89 (84.8)	0.739
Genetic mutations	6 (66.7)	25 (23.8)	0.007^*^

* *Statistically significant*.

### Post-operative Follow-Up

The median follow-up of 46 pediatric patients was 28.5 months, with a clinical remission rate of 87% in pediatric patients with PD (Table 6). The median number of acute pancreatitis episodes decreased from four per year (1–10/year) pre-operatively to one per year (1–3/year) post-operatively (*P* < 0.001). Twenty-four (70.83%) children had a complete remission (no abdominal pain and acute pancreatitis) post-operatively; among them, seven (7/18) children with ARP and 17 (17/28) with CP had complete remission after an average follow-up of 15.71 and 26.12 months, respectively. Although no scale assessment was performed, most of the children indicated on follow-up that the severity of the attack was lessened and could be alleviated by a low-fat diet at home, not requiring hospital care. During long-term follow-up, there was an increase in post-operative BAZ post-operatively (0.04 ± 1.16) in patients relative to their pre-operative BAZ (−0.42 ± 1.15) (*P* = 0.004). In the pediatric patients with CP, clinical remission rate was 87.9%, and the number of post-operative episodes of pancreatitis were significantly reduced that of pre-operative episodes (*P* < 0.001). Moreover, there was an increase in BAZ post-operatively (0.04 ± 1.24) relative to pre-operative BAZ (−0.57 ± 1.24) (*P* = 0.003). Six (13.0%) pediatric patients with CP had pre-operative malnutrition (BAZ: −2.49 to −2.02), of whom four had improved nutrition after surgery by catch-up growth at a mean follow-up of 10.75 months (BAZ: −1.93 to 1.09), while the other two did not show any significant worsening.

**Table 6:** PEP impact of female, genetic mutations, and stone extraction on the risk of post-pancreatitis.

	***P***	**OR (95%CI)**
Female	0.997	0.000 (–)
Genetic mutations	0.064	0.061 (0.003, 1.177)
Stone extraction	0.229	6.575 (0.305, 141.781)

## Discussion

There are few studies on the application of ERCP in children with PD, and its efficacy and safety in the pediatric population are yet to be elucidated. In this study, we selected children with symptomatic PD who were treated with ERCP and analyzed their basic information, the ERCP procedure, operational complications, PEP risk factors, and long-term follow-up results.

Forty-six pediatric patients with PD were included in this study, and 114 ERCP operations were performed. The median age at PD onset 9 years, and there were 22 woman and 24 men, with an even distribution of PD by sex and age. There were 13 (39.13%) and 33 patients (60.87%) with ARP and CP, respectively. Twenty-six pediatric patients completed genetic testing, 15 of whom had gene deletions and no history of hypertriglyceridemia, smoking, or drinking.

The concept of PD as a cofactor rather than a single cause is gaining recognition. Increasing studies have confirmed that PD is a pathological basis for pancreatic disease and that pancreatitis only occurs when PD is combined with risk factors such as gallstone disease, overeating, alcohol consumption, hypertriglyceridemia, and genetics (4). Genetic abnormalities, such as SPINK1, PRSS1, and CFTR gene mutations, are the main risk factors for symptomatic PD in children, and these genetic variants lead to the blockage of the pancreatic duct due to highly viscous pancreatic secretions, inducing pancreatitis in children with PD (5, 6). The gene detection rate (57.7%, 15/26) was high in children with PD in this study, but no risk factors, such as smoking, alcohol consumption, or hypertriglyceridemia, were found. It was hypothesized that genes might synergize with PD to cause the development of pancreatitis. The predominance of the SPINK1 variant (73.3%, 11/15) was consistent with the findings of Wen et al. (7) in Shanghai, China. However, INSPPIRE (4) reported CFTR variants were the most predominant ones (37%), followed by SPINK1 (30%). The difference in gene variants might have been caused by the ethnic differences.

Regardless of the mechanism, the obstruction of the lesser papillae appears to be central to the pathogenesis of symptomatic PD (8). Thus, the pressure gradient in the lesser papillae can be reduced surgically or endoscopically. Liao et al. (9) reviewed the literature on endoscopic treatment or surgery for PD in adults from Medline, including 15 endoscopic treatments and 13 surgical treatments, and compared the efficiency of the two modalities in PD treatment. They found that the ERCP treatment efficiency (69.4%, 361/520) and surgical treatment efficiency (74.9%, 203/271) were similar (*P* = 0.106), although ERCP had the advantages of shorter anesthesia duration, fewer complications, and shorter hospital stay. Given the incomplete development of organ function and the poor tolerance to surgical trauma in children, most studies recommend ERCP as the standard treatment of choice for symptomatic PD in children, and parents similarly prefer endoscopic treatment (10). In the present study, which included 46 pediatric patients, the ERCP treatment modalities used were the same as in adults, mainly endoscopic papillotomy (87.0%), pancreatic duct stenting (91.3%), and followed by pancreatic duct dilation (63.0%) and stone extraction (73.9%). The post-ERCP clinical remission rate was 87%, with fewer post-ERCP episodes of pancreatitis (*P* < 0.001) and significantly improved nutritional status (*P* = 0.004). The post-ERCP complication rate and PEP rate were 10.5 and 7.9%, respectively, with improvement and no deaths, indicating that ERCP is effective and safe in the treatment of PD in children.

Most reports suggest that adults with ARP benefit more from ERCP treatment than those with CP (11) and that in patients with CP, irreversible pancreatic duct lesions (e.g., pancreatic duct dilation and pancreatic duct stones) affect drainage despite sphincterotomy and/or stent placement (12, 13). However, it has also been revealed that patients with CP can also benefit from ERCP. Bhasin et al. (14) found that patients with CP with pancreatic duct dilation had an ERCP treatment efficiency of 95%. Lin et al. (4) reported an ERCP treatment efficiency of 83% in the presence of pancreatic duct stones in CP. All the reports retrieved from Medline on ERCP for PD in children concluded that children with CP also benefit from ERCP (4, 15–17). In the present study, the ERCP treatment efficiency for CP was 87.9%, with a significant reduction in the number of pancreatitis episodes and a significant improvement in nutritional status after the operation. A reason that children with CP seem to respond better to ERCP treatment than adults may be that CP is a chronic, progressively exacerbating process. Children with CP are still in the early stages of the disease and have less severe pancreatic inflammatory damage than adults, them respond better to ERCP treatment than adults.

PEP is the most common complication of ERCP. For adults, 100 mg diclofenac or indomethacin is recommended before ERCP, but there are no clear recommendations for children undergoing ERCP. Thus, no medications were pre-operatively administered in our study. Pancreatic duct injection, pancreatic sphincterotomy, non-prophylactic pancreatic duct stenting, operator experience, and the female sex have been identified as the risk factors for PEP (18, 19). Michailidis et al. (12) performed a meta-analysis of 23 publications and found that the incidence of PEP was 10.1% among 874 patients with PD who were treated with ERCP, which was higher than that in other indications. This was likely due to the high technical difficulty of small papillary cannulation and damage to the pancreas. In contrast, Meng et al. (20) that among 187 patients with PD, and the occurrence of PEP in patients with CP was significantly lower than that in non-CP-type PD (5.6 and 15.7%), suggesting that CP is a protective factor against PEP. Whether prophylactic pancreatic stenting prevents PEP remains debatable. Choudhary et al. (21) concluded that pancreatic duct stenting is a protective and reduces the incidence of PEP in adults, and another study (22) concluded pancreatic duct stenting reduces the incidence of PEP in non-CP patients, but is not preventive for PEP in CP patients. The incidence of pancreatitis after our ERCP procedure was 7.9%, which was lower than the previously reported incidence in our other indications (20.7%) (23). Risk factor analysis of sex, age, CP, genetics, and mode of operation (e.g., pancreatic duct stenting, stone extracting, pancreatic duct dilation, and papillotomy) indicated that the female sex, stone extraction, and genetics may be risk factors, but were not statistically significant. Stenting was neither protective nor a risk factor for PEP.

This study has certain limitations. First, this is study a retrospective study with potential biases in inclusion criteria. Second, it is a single-center study with a small sample size and insufficient overall follow-up time; thus, the long-term efficacy of ERCP and the morphological changes in the pancreatic duct after ARP were not assessed.

In conclusion, therapeutic ERCP is feasible in children with symptomatic PD as it shows a high clinical remission rate, contributes to nutritional status recovery, and improves the quality of life of the pediatric patients, with manageable and few post-operative complications. Further large, multicenter investigations on the efficacy and safety of ERCP for PD in children are necessary to validate the present study findings, and the long-term treatment effects and structural morphological changes of the pancreatic duct need to be assessed.

## Data Availability Statement

The original contributions presented in the study are included in the article/supplementary material, further inquiries can be directed to the corresponding authors.

## Ethics Statement

The study protocol was approved by the Ethics Committee of Shanghai Children's Medical Center (SCMCIRB-K2019005). All the participants' legal guardians provided written informed consent.

## Author Contributions

BG and ZD designed the study. GP acquired, analyzed, and interpreted the data. GP and KY drafted the manuscript. ZD edited the manuscript. All authors contributed to the article and approved the submitted version.

## Funding

This work was supported by grants from the Shanghai Municipal Health Commission of China [Grant Numbers: 2018LP018 and ZY(2018-2020)-FWTX-1105].

## Conflict of Interest

The authors declare that the research was conducted in the absence of any commercial or financial relationships that could be construed as a potential conflict of interest.

## Publisher's Note

All claims expressed in this article are solely those of the authors and do not necessarily represent those of their affiliated organizations, or those of the publisher, the editors and the reviewers. Any product that may be evaluated in this article, or claim that may be made by its manufacturer, is not guaranteed or endorsed by the publisher.
